# Sexual Neurasthenia

**Published:** 1884-07

**Authors:** 


### SEXUAL NEURASTHENIA, ITS HYGIENE, CAUSES, SYMPTOMS, AND TREATMENT, WITH A CHAPTER ON DIET FOR THE NERVOUS. By George M. Beard, A. M., M. D. Edited by A. D. Rockwell, A. M., M. D. Pp. 270; price, $2.00. New York: E. B. Treat, 1884.

Doctors Beard and Rockwell are so well known in connection with the study and treatment of nervous troubles that any work from them is sure of respectful attention. The present volume is composed of posthumous manuscript left by the late Dr. Beard and edited by Dr. Rockwell. The work is novel in its idea and interesting throughout; especially so to us is the chapter on "Diet for the Nervous." Dr. Beard believes in the evolution theory and would apply it to foods. He writes: "1st. The earth feeds on grass; fruits and cereals feed on the earth; the lower animals feed on fruits and cereals and on other animals; man, therefore, should feed mainly on the lower animals, with a small proportion of fruits and cereals."

"2d. In proportion as man grows sensitive through civilization or through disease, he should diminish the quantity of cereals and fruits, which are far below him in the scale of evolution, and increase the quantity of animal food, which is nearly related to him in the scale of evolution, and therefore more easily assimilated."

Moreover, it is stated "that man *is* good for man; and cannibals *are* the strongest and healthiest of savages."

Although the therapeutic measures recommended in this volume are different from those we of the Hahnemannian school rely on, nevertheless, the volume is well worth a careful perusal.

